# Supplementary material for: Imaging in Diagnosis of Systemic Sclerosis
Source: J Clin Med. 2021 Jan 12;10(2):248. doi: 10.3390/jcm10020248 (PMC7827740; doi:10.3390/jcm10020248)
Supplement: Supplementary file 1 [file jcm-10-00248-s001.zip › jcm-1032375-supplementary/jcm-1032375-supplementary.docx]

Table S1: Summary of indications and pitfalls for various imaging modalities in different clinical forms of systemic sclerosis.

| **Area** | **Indications and findings** | **Pitfalls and comments** |
| --- | --- | --- |
| **CLASSICAL RADIOLOGY** | | |
| **Musculoskeletal system** | Preliminary assessment of acro-osteolysis, calcifications and soft-tissue thinning (plain radiograph) | Differentiation between rheumatoid arthritis and SSc might be difficult |
| **Respiratory tract** | Preliminary assessment of pulmonary fibrosis (plain radiograph) | Pulmonary fibrosis is visible only in advanced stage |
|  | Confirmation of PAH  (right heart catheterization) | Invasive procedure |
| **Gastro-intestinal tract** | Evaluation of the esophageal motility  (videofluoroscopy) | Administration of barium sulfate might exacerbate small bowel related symptoms - iodine swallow should be considered |
|  | Evaluation of the delayed voiding of the stomach radiography using radiopaque pellets |  |
| **ULTRASOUND** | | |
| **Skin** | Measurement of skin thickening |  |
|  | Distinguishing edematic lesions from fibrosis | Reproducibility is relatively low |
|  | Quantitative skin fibrosis assessment  (shear-wave elastography) |  |
| **Musculoskeletal system** | Thickening of the tendon sheaths |  |
|  | Increased tissue blood flow due to articular inflammation (Color Doppler) |  |
| **Respiratory tract** | Accessory examination method in diagnosing pulmonary fibrosis |  |
| **Cardiovascular system** | Preliminary examination in evaluation of PAH and right ventricular (RV) dysfunction |  |
|  | Assessment of myocardial tissue velocities (Spectral Doppler echocardiography) |  |
|  | Accelerated atherosclerosis in peripheral arteries | Ambiguous study results regarding accelerated atherosclerosis in scleroderma |
| **COMPUTED TOMOGRAPHY** | | |
| **Respiratory tract** | Evaluation of interstitial lung disease  (HRCT) | Findings similar to non-specific interstitial pneumonia (NSIP) |
|  | Excluding pulmonary embolism in case of acutely deteriorating respiratory function  (CT angiography) |  |
|  | Symptoms of PAH |  |
| **Gastro-intestinal tract** | Esophageal dismotility | Usually additional findings in HRCT of the chest |
|  | Ectasia of the antral part of the stomach |  |
|  | Fibrosis in the small bowel with dilation (>3cm) |  |
| **Heart** | Pericardial effusion and pericardial fibrosis | Usually additional findings in HRCT of the chest |
| **MAGNETIC RESONANCE IMAGING** | | |
| **Musculoskeletal system** | Differentiation between fibrosis and edema |  |
| **Heart** | Evaluation the morphology, function and vitality of the cardiac muscle |  |
|  | Visualisation of indirect symptoms of fibrosis and direct assessment of the muscular involvement |  |
|  | Differentation between myocardial fibrosis and edema (T1 and T2 mapping and LGE) |  |
